# Rapid Innovation in Diabetes Care During Covid-19

**DOI:** 10.1177/2633559X20951168

**Published:** 2020-11

**Authors:** Jessica Odom, Celia Beauchamp, Casey Fiocchi, Meredith Eicken, Michelle Stancil, Jenn Turner, John Bruch

**Figure:**
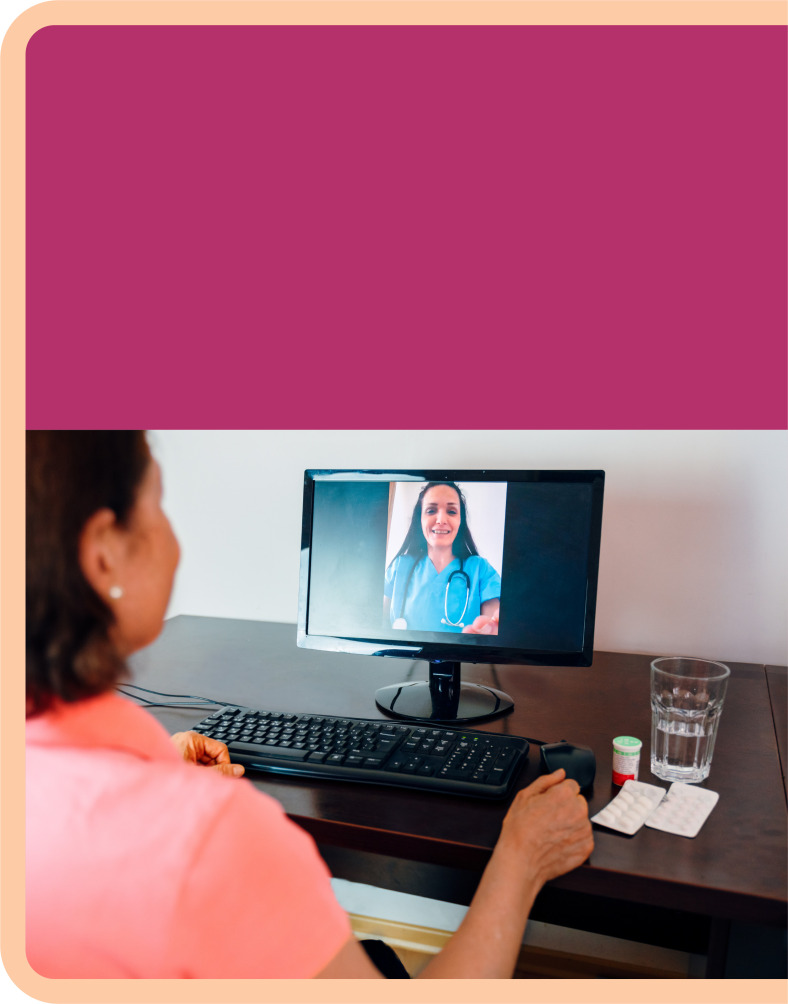


Prisma Health-Upstate’s Diabetes Self-Management Education and Support (DSMES) program began over 20 years ago with 3 employees. Currently, the program has 25 diabetes care and education specialists (DCES), including 8 registered dietitians (RD), 13 registered nurses (RN), a licensed clinical social worker (LCSW), 3 pharmacists, and 4 support staff. In all, 84% of the staff hold the credential of Certified Diabetes Care and Education Specialist (CDCES) or Board Certified in Advanced Diabetes Management (BC-ADM).

The Prisma Health-Upstate DSMES program is recognized across the state of South Carolina and the nation as one of this country’s leading diabetes care and education programs. It is a robust and consistent program that serves participants of all ages at 13 sites across 6 counties. In 2019, the program received an average of 408 referrals per month and served 1701 participants. The program achieves an average A1C reduction of 1.14% compared to the national average of 0.6%.^1^

Prisma’s DSMES program expanded in 2019, adding 3 locations, and it received 10% more referrals that year than in the previous year. This American Diabetes Association accredited DSMES program was poised to continue its growth caring for participants with diabetes in the upstate of South Carolina when in March 2020, COVID-19 began to affect the state.

## Meeting the COVID-19 Moment

Due to COVID-19, the traditional services offered by Prisma’s DSMES program to people with diabetes stopped. Like many outpatient programs, Prisma’s parent site and 7 of its 9 expansion sites were closed. This necessitated rapid-cycle innovation to maintain provision of care to participants with diabetes. This was particularly important in South Carolina, where 13% of the adult population has diabetes.^2^

Early data from the Centers for Disease Control and Prevention found that patients with underlying chronic conditions, like diabetes mellitus, were at increased risk for severe cases of virus.^3^ This underlying condition was often cited as a contributor in the death of people infected with COVID-19.

To care for this high-risk population, our multidisciplinary team quickly adapted and created innovative, technology-based solutions to allow safe and timely DSMES to be provided during mandatory social distancing initiated during the pandemic.

## Virtual Visits

The electronic health record (EHR) portal allows video visits with providers via smartphone, computer, or another device. When participants are scheduled, assessment questionnaires are assigned that can be opened and completed in the portal prior to the visit. The day before the visit, participants are preregistered by a DSMES staff member with prior business office experience. Participants are informed of their benefits for DSMES or medical nutrition therapy (MNT) and any anticipated patient financial responsibility.

The specialist sends a reminder message to the participant 1 or 2 days prior to the visit. Electronic copies of educational materials specific to the visit are attached to the message and can be printed or accessed electronically prior to and during the visit. Some care and education specialists work remotely from home; others work from DSMES offices and are able to maintain social distancing with limited staff in the buildings.

At the scheduled time of the visit, both the patient and provider log on remotely. On the schedule, there is a green icon to let the provider know the participant has connected via video. Participants are asked if they consent to receive education via video, and their name and date of birth are confirmed. Any additional attendees are identified and documented.

If the participant is unable to access the EHR portal or the audio or video is not working, alternative video conferencing platforms such as Skype or Doxy.me can be used. Participants must be informed that privacy and security of these platforms cannot be assured, and they must verbally consent to continue the visit.

Prior to the pandemic, electronic questionnaires were utilized by the program to track participant data and outcomes. Information such as the percentage of time the participant followed a healthy eating plan, took diabetes medication, tested their blood sugar, and exercised is collected. In addition, participants can provide feedback on the program and the education provided. A current A1C is requested. This information is used to improve the program, better meet the needs of participants, and collect postprogram data.

## Diabetes Toolkit

To facilitate virtual education, participants needed to have access to the EHR patient portal on their smart device or personal computer. In addition, no diabetes providers were currently conducting virtual visits. Navigating these services was sometimes challenging. Therefore, a diabetes toolkit was developed to rapidly train clinical team members on how to conduct a virtual visit and improve the patient experience.

Tip sheets were developed by the organization and department staff to provide DCES and support staff with information on how to sign participants up for EHR services, provide a virtual visit, troubleshoot sound and technical difficulties, and send pre-visit messages to help prepare for visits. Diabetes education handouts were organized and placed on a shared drive to give providers quick access. Step-by-step instructions were developed to assist team members with scheduling, attaching patient questionnaires, and sending materials and instructions to participants. Smart phrases for accurate documentation were developed for each visit type. The smart phrases include required telehealth documentation for CMS and insurance billing.

The toolkit was assembled into notebooks for each diabetes provider to use during the video visit. Additionally, an insulin instruction video used for inpatient teaching was incorporated into the outpatient curriculum toolkit.

## Staffing Adjustments

The rapid decrease of in-person DSMES encounters required a quick evolution of staffing needs and billable opportunities. Only RDs and LCSWs are currently approved by the Centers for Medicaid and Medicare Services (CMS) as telehealth providers for billing purposes. Although DCES were already providing remote monitoring across adult, pediatric, and obstetrical populations, DSMES was not billing for telehealth encounters prior to March 2020.

To maximize the available workforce, staffing was restructured to utilize RDs and the LCSW as revenue-generating, virtual visit providers.

To maximize the available workforce, staffing was restructured to utilize RDs and the LCSW as revenue-generating, virtual visit providers. Because RNs and pharmacists were not authorized to bill for telehealth visits before August 2020, these providers were strategically moved to the remaining open locations to provide essential in-person visits that were unable to be provided remotely. Essential visits included pregnancy and diabetes, insulin starts, insulin pump and sensor training, basic diabetes education to those newly diagnosed, and other visits as requested by providers. These essential visits continued to be provided in person at a centrally located expansion site, a pediatric endocrinology office, and an obstetric clinic. One inpatient care and education specialist staffed the flagship hospital. During the pandemic, RN and pharmacist DCES also continued normal operations providing remote monitoring services to over 100 of the program’s most vulnerable adult, elderly, pediatric, and pregnant participants. Remote monitoring helps participants to self-manage their diabetes and connects them to their medical providers as needed.

Participants that required interpreter services were initially scheduled as nonbillable telehealth visits with nurses or pharmacists. Later, interpreters were included in a 3-way video visit that allowed for billing. Tip sheets provided to staff aided in the addition of this service to participants.

Although a few team members needed to be reoriented to work at new locations, many staff were already working across different populations (eg, pediatrics, OB). All DCES routinely complete annual medication competencies for adult, pediatric, and obstetric populations, which made it easier for them to transition into new roles and locations.

## Results

The show rate for video visits was very high; 86% mid-March to mid-April 2020 compared to 82% for in-person visits mid-February to mid-March 2020 – prior to the pandemic.

The show rate for video visits was very high; 86% mid-March to mid-April 2020 compared to 82% for in-person visits mid-February to mid-March 2020— prior to the pandemic. In the initial 6 weeks, 220 virtual visits were completed in the EHR portal with an 84% success rate.

Participants enjoyed the experience and were eager to have the visit rescheduled when either personal, video, or audio issues interfered with the completion of a scheduled visit. For participants in rural areas, where transportation and child care are an issue, and for those who work during the day, video visits were much appreciated and well attended. DCES have provided feedback that many participants were eager to set up subsequent virtual visits based on their satisfaction with the service and its convenience.

**Figure:**
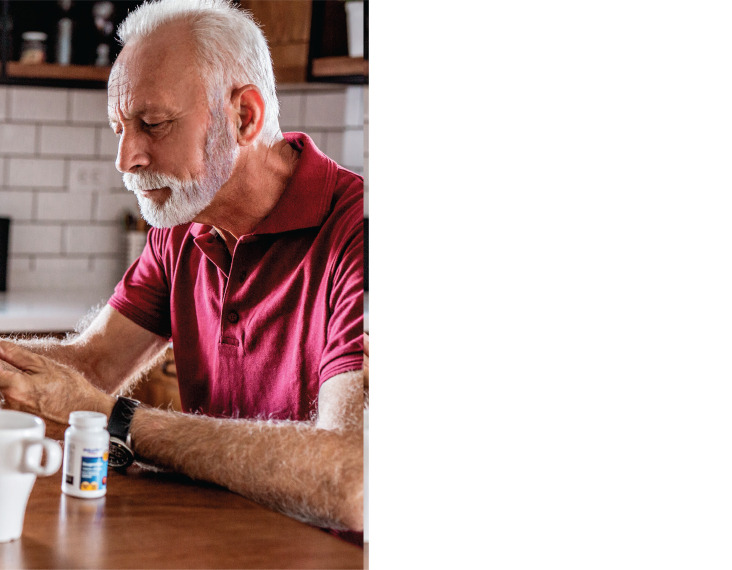


## Challenges

Participants and staff needed to be taught how to access video visits via the EHR because visits up until this time were conducted in person due to reimbursement requirements. Much of the training was “learn as you go.” Many participants did not understand the technology or had never used it before. Prisma Health quickly set up a help line to provide technical assistance to participants accessing virtual visits.

Internet access and bandwidth issues caused some difficulties in the video and audio portions of the visit, depending on what services participants had available to them. Often care and education specialists were required to make quick decisions when technology malfunctioned. Tip sheets were developed to help staff navigate the visit and assist participants with problem solving when technical problems occurred. In addition, the department assigned a clinical care and education specialist with super-user skills to provide at-the-elbow services to staff daily.

Although one of the biggest challenges was technology, initial concerns also included the following:

- What do we do if a participant does not have
- cell phone or computer access?
- Will the participant show up for the visit?
- Will the sound and visual quality of the visit be good?
- Will there be too many interruptions from the home during the visit?
- Will the participant open the pre-visit message with the handouts and assessment questionnaire?
- How can a care and education specialist know enough to trouble shoot the technical issues that might come up?
- How many visits can a care and education specialist complete in a day?

Many of the early concerns did not turn out to interfere with the delivery of services or the quality of patient education and care. DSMES continues to provide video visits and revise procedures as new updates and guidelines are received.

## Conclusion

With rapid innovation and a dedicated team, Prisma Health-Upstate DSMES transformed to meet the COVID-19 moment. People with diabetes continued to receive care, DSMES staff continued to work at 80% normal capacity, and billable visits generated revenue for the department. In line with the health system’s actions, as-needed (PRN) employees were not utilized during the initial months of the pandemic due to financial constraints.

Virtual visits overcome barriers of transportation, childcare, work schedules, and other obstacles that prevent participants from accessing DSMES.

As all health care providers move forward in these uncertain times, there may be a silver lining in a time of crisis. Virtual visits overcome barriers of transportation, child care, work schedules, and other obstacles that prevent participants from accessing DSMES. Even as traditional services reopen in the future, Prisma Health DSMES will continue to offer both in-person, virtual, and remote monitoring services to provide patient-centered care, meeting the patients where they are in their journey with diabetes.
